# Validation of a Chinese Positive Youth Development Scale: Dimensionality and factorial invariance

**DOI:** 10.1371/journal.pone.0303531

**Published:** 2024-05-31

**Authors:** Xiaoqin Zhu, Carman K. M. Chu, Xue Wu, Daniel T. L. Shek

**Affiliations:** 1 Department of Applied Social Sciences, The Hong Kong Polytechnic University, Hong Kong, PR China; 2 Office of Undergraduate Studies, The Hong Kong Polytechnic University, Hong Kong, PR China; Aalborg University, DENMARK

## Abstract

For a multi-dimensional measure of positive youth development (PYD), its factor structure should be invariant across groups and over time. This study examined the factorial validity of the 44-item short form of the “Chinese Positive Youth Development Scale” (CPYDS-SF) that assesses 15 dimensions of PYD attributes. Using two waves of longitudinal data with a one-year interval in between, this study examined the factor structure of the scale and whether the structure is invariant between gender groups and across time. The data were collected from 3,328 adolescents at Wave 1 and 3,638 adolescents at Wave 2, with a matched sample of 2,905 adolescents (mean age = 12.57 ± 0.72 at Wave 1; 49.54% girls). Confirmatory factor analysis revealed that the 15-factor structure fitted the data well. The findings of invariance tests further supported this structure’s invariance across gender and time, indicating a stable factor structure of CPYDS-SF among Chinese adolescents. These findings suggest that CPYDS-SF can be used to examine gender differences and the longitudinal development of PYD qualities among Chinese adolescents.

## Introduction

Adolescence is a critical period characterized by higher risks of mental illness and problematic behaviors [1–3]. The traditional pathology-based perspective attempts to “eliminate” adolescent developmental problems by dealing with specific risk factors of the respective problem [4]. This approach has been criticized for merely focusing on single problematic behavior while ignoring the co-occurrence of multiple problematic behaviors, and for the wrong assumption that the absence of problems means good development [1]. With a paradigm shift from focusing on pathologies to strengths, the positive youth development (PYD) approach posits that adolescents have potentials to thrive through building multi-faceted developmental assets (e.g., internal and external PYD qualities), which would reduce the likelihood of engaging in problem behaviors while simultaneously increasing the likelihood of having positive growth and well-being [1, 5, 6]. In line with this theoretical proposition, empirical findings have established the close associations between PYD qualities and better developmental outcomes, such as better mental health, higher well-being, and fewer problematic behaviors [e.g., 7–9]. As Humphrey, Kalambouka [9] have argued, there has been a growing interest and investment in promoting PYD qualities, such as social and emotional skills, as a means to promote children’s well-being, adjustment, and academic achievement. Indeed, rich evidence has shown the benefits of promoting PYD qualities through effective youth program in enhancing life satisfaction, thriving, learning engagement, academic achievement, and prosocial behavior as well as in reducing substance abuse, juvenile delinquency, aggressive behaviors, and academic failure [6, 10, 11].

Multiple frameworks have been proposed to conceptualize PYD qualities [1]. For example, the “Five Cs” model emphasizes “*Competence*,” “*Confidence*,” “*Character*,” “*Connection*,” and “*Caring*” as outcomes of positive individual adjustment to contextual factors, which further lead to the “six C,” namely “*Contribution*” to self, others, groups, family, community, and society [5]. While achieving these “six Cs,” adolescents are also simultaneously less likely to suffer from dysfunctional development [e.g., 12]. Another framework is “social and emotional learning” (SEL), which concentrates on five dimensions of SEL competencies, including “self-awareness,” “social awareness,” “self-management,” “relationship skills,” and “responsible decision making” [13]. Numerous evaluation studies have demonstrated the contribution of cultivating these qualities in promoting youths’ healthy functioning and well-being [1, 10].

In addition, based on an inductive framework, Catalano et al. [14] identified 15 PYD constructs from effective youth prevention programs. The fundamental thesis is that enhancement programs cultivating 15 PYD qualities among adolescents are effective in promoting their positive outcomes while reducing negative outcomes. These qualities consist of “cognitive competence,” “behavioral competence,” “self-determination,” “prosocial norms,” “prosocial involvement,” “belief in the future,” “clear and positive identity,” “bonding,” “social competence,” “emotional competence,” “moral competence,” “resilience,” “self-efficacy,” “spirituality,” and “recognition for positive behavior” (see Table 1 for definitions of these qualities). This PYD framework covers the essential elements intrinsic to positive youth development that includes a broad array of qualities, including psychological, social, emotional, behavioral, moral, and cognitive competences. The benefits of PYD attributes have been supported by fruitful empirical evidence [11, 15]. More importantly, this framework integrates essential PYD concepts highlighted in the “Five Cs” (e.g., different psychosocial competencies including moral competence, self-worth, and prosocial involvement) and SEL (i.e., competencies in cognitive, emotional, social, and behavioral dimensions) models [16].

**Table 1:** Definitions of the 15 PYD qualities and sample items.

Four higher-order dimensions	The 15 basic PYD qualities	A brief definition of the construct	Sample item in the scale
Cognitive-behavioral competence	1. Cognitive competence	Intellectual skills such as critical thinking and problem-solving.	I try new ways to solve my problems.
	2. Behavioral competence	Behavioral skills such as taking verbal and non-verbal actions.	I can express views that are different from others.
	3. Self-determination	Abilities to take age-appropriate actions with independent thinking	I am able to make wise choices.
Prosocial attribute	4. Prosocial norms	Clear standards for prosocial engagement.	I care about unfortunate people in society.
	5. Prosocial involvement	Engagement in activities to make positive contributions.	I will try my best to contribute to my school or society.
Positive identity	6. Beliefs in the future	Being optimistic about one’s future and able to develop future goals and options.	I have the confidence to solve my future problems.
	7. Clear and positive identity	Having positive and healthy self-perceptions.	I am a person with self-confidence.
General PYD quality	8. Bonding	Being able to establish positive relationships with positive peers and healthy adults.	When I need help, I trust my teachers will help me.
	9. Social competence	Interpersonal skills such as communication and identification with groups.	I know how to communicate with others.
	10. Emotional competence	Abilities to recognize and manage emotions.	When I am unhappy, I can appropriately show my emotions.
	11. Moral competence	Abilities to make sound moral judgments and behave accordingly.	I have high moral expectations about my behavior.
	12. Resilience	Abilities to adapt to and deal with adversity in positive and effective ways.	When I face difficulty, I will not give up easily.
	13. Self-efficacy	A belief that one has abilities and can attain goals.	I can finish almost everything that I am determined to do.
	14. Spirituality	Sense of purpose and meaning in life and hope.	My life is exciting.
	15. Recognition for positive behavior	Having contexts that reward, recognize, or reinforce young people’s positive behavior.	When I help others, my classmates will recognize my behavior.

Researchers have adopted different measures to assess PYD qualities. For example, twelve different assessment tools were identified in Humphrey et al.’s [9] systematic review on measures of SEL skills in children and young people. Multiple scales have also been applied in measuring “Five Cs” in different youth samples [e.g., 17–19]. Obviously, valid and reliable assessment tools on PYD qualities are of paramount importance in the evaluation of PYD programs since it allows a more precise assessment of group differences or overtime changes in PYD qualities [20]. Unfortunately, existing literature regarding the assessment of PYD qualities showed several limitations. First, most of the extant PYD measures are developed in Western contexts based on Western samples, particularly North American young people. We argue that assessment tools with good reliability and validity among Western youth may not necessarily work effectively in non-Western populations, such as Chinese adolescents, due to the possible distinctive interpretations and structures of PYD qualities [21, 22]. Furthermore, the studies conducted in the Hong Kong sample did not report reliability values [23].

Second, although factor analyses have been used to examine the different dimensions underlying measures of PYD attributes, there are several problems. Primarily, some studies only used exploratory factor analyses [e.g., 24, 25]. Moreover, amongst studies using confirmatory factor analyses, very few have further evaluated whether a scale’s factorial structure is stable across subgroups (e.g., boys vs. girls) through invariance tests. For example, Phelps et al. [19] used a 78-item scale to measure “Five Cs” among participants attending a summer sports camp. The authors tested the factor structure of the scale without examining factorial invariance. Similarly, Lopez et al. [18] developed the “Bridge-Positive Youth Development Scale” based on the “Five Cs” model and examined the scale’s structural properties without testing invariance. In developing measurement instruments, it is crucial to take into account respondent subgroups that exhibit distinct features, such as gender groups, as they may conceive a concept (e.g., PYD quality) in different ways [26]. In particular, gender differences in youth development have been widely studied and acknowledged in the field of Psychology and Development Science. Research has shown that males and females may experience distinct developmental trajectories, face different challenges, and exhibit varying patterns of strengths and vulnerabilities during adolescence [27–30]. For example, connection appeared to be of greater importance for girls than boys. Whereas girls’ connection was associated with less somatic anxiety, fewer physical and psychological symptoms, and high well-being, boys’ connection was only a predictor of high well-being [30]. However, boys who experienced higher ACEs were at a heightened risk of exhibiting elevated externalizing trajectories [28]. Nevertheless, most of the studies failed to establish the measurement equivalence of the measures across genders to rule out the possibility that gender differences are in fact an artifact of measurement differences. Thus, testing measurement invariance across groups (e.g., gender groups) plays an important role in ensuring the same structural interpretation between groups, such that the scores derived from the assessment tools can be meaningfully compared between groups [31].

Third, while there is an increasing emphasis on longitudinal invariance of valid assessment tools [32, 33], very few studies have examined the longitudinal invariance of PYD measures. For example, the PYD measure developed by Wen et al. [34] based on the “Five Cs” model showed factorial invariance across gender (e.g., boys vs. girls) and geographical location (e.g., rural vs. urban) in Chinese contexts. However, its longitudinal invariance is unknown. Chai et al. [22] further examined the scale’s psychometric properties and found that the “Four Cs” structure without the “*caring*” dimension better fit the data among Chinese adolescents. The authors argued that elements underlying “*caring*” can be accounted for by the love under “*character*” or the relationship with others under “*connection*” due to the collectivistic social structure in mainland China. Nevertheless, the authors did not examine longitudinal measurement invariance of the four-factor structure either.

While cross-group invariance enables cross-group comparisons of PYD qualities, longitudinal factorial invariance sets the foundation for over-time comparisons by ensuring that a given scale steadily measures the same construct with an identical structure on different occasions [35, 36]. This proposition has been increasingly acknowledged in recent years. For example, recent youth research has examined factorial invariance over time for different measurements, such as the “Problematic Trait Inventory” [37], the “Short Grit Scale” [38], and the “Center for the Epidemiological Studies Depression Scale” (CES-D) [32, 33]. Specifically, the longitudinal invariance of the CES-D ensures that any changes derived from the scale between different time points are attributable to over-time development rather than structural instability in measurement [33].

Given considerable developmental changes in physiological, intellectual, psychological, and social domains over the adolescent period, adolescents’ interpretations and perceptions of their personal attributes, including PYD qualities, may vary over time. Noteworthy, age differences in PYD have also been widely acknowledged in previous studies [39–41]. This requires testing of longitudinal validity of a certain PYD scale to make sure that any longitudinal differences derived from the scale are due to life span development rather than measurement error over time [42, 43]. As emphasized by Putnick and Bornstein [44], analyses of measurement invariance over time become particularly important when researchers aim to evaluate program effectiveness by comparing pretest and posttest scores. The key is that the lack of longitudinal invariance will make the comparisons meaningless. Therefore, it is necessary to examine the longitudinal invariance of assessment tools on PYD attributes.

Within different Chinese contexts, Shek and his collaborators developed the 80-item “Chinese Positive Youth Development Scale” (long form, CPYDS-LF) based on the 15 PYD constructs identified by Catalano et al. [14]. This scale measures 15 PYD qualities (e.g., bonding and spirituality) using 15 subscales. According to Shek and Ma [45], the CPYDS-LF possessed adequate factorial validity and internal consistency and the 15 subscales can be further subsumed into four higher-order dimensions, including “cognitive-behavioral competencies,” “prosocial attributes,” “positive identity,” and “general PYD qualities” (see Table 1). In addition, invariance tests indicated that the four-higher-order-factor structure was invariant across gender groups. Given its good validity and reliability, the scale has been widely applied in assessing the impacts of a multi-year PYD program entitled “P.A.T.H.S. Project” and investigating the relationship between PYD qualities and Chinese adolescents’ developmental outcomes [15, 46].

To reduce scale length and minimize respondents’ fatigue in completing the questionnaire, a 44-item version of CPYDS (i.e., short form, CPYDS-SF) has also been created by selecting the three items having the highest loadings in each construct (two items in the self-efficacy subscale as there were in total two items in the original scale). Constructing CPYDS-SF as a short form the original CPYDS can contribute to youth research in several aspects. First, a short form allows for a more efficient and streamlined assessment process. Adolescents are often faced with various demands and time constraints in the classroom context, and a shorter assessment tool can minimize their burden and increase participation rates. This is particularly important in longitudinal studies where repeated measurements are required over time. Additionally, a short form can enhance the scalability and practicality of the PYD assessment. It can be easily administered in various settings, such as schools or community organizations, where resources and time may be limited. The availability of a concise measure facilitates the integration of PYD assessment into existing research or intervention programs, promoting the widespread implementation of PYD principles in practice. Furthermore, a short form can contribute to the dissemination of research findings and facilitate cross-study comparisons. When researchers adopt a standardized short form, it enhances the comparability of results across different studies [17, 23, 47–49]. This comparability strengthens the evidence base and allows for a more comprehensive understanding of PYD qualities among diverse populations and contexts. Lastly, the development of a short form reflects the ongoing refinement and advancement of measurement instruments in the field of PYD. By identifying the key dimensions and items that capture the essence of PYD, researchers can gain insights into the core components that contribute to positive youth outcomes in a more parsimonious manner. This knowledge can inform the design and implementation of targeted interventions and programs aimed at promoting PYD.

While the CPYDS-SF has also been widely adopted in youth research [46, 50, 51], no validation research has been conducted to examine the factorial validity and its invariance in different groups (e.g., gender) and over time. Given the above research gaps, the present study aimed to test the factor structure of the 44-item CPYDS-SF among Chinese adolescents. In addition, we also tested whether the factor structure was invariant across gender and over time. By validating and testing the measurement invariance of CPYDS-SF, we aimed to contribute to the existing literature by constructing a valid and reliable assessment tool for PYD qualities, which can be meaningfully adopted to investigate gender differences and longitudinal changes.

## Materials and methods

### Participants and procedures

The current research used two waves of data collected in a longitudinal youth project in Hong Kong, which aimed to assess Hong Kong Chinese adolescents’ developmental outcomes and related psychosocial correlates. A list of 399 public local secondary schools was formed based on information provided by the Education Bureau. A total of 30 schools in different districts were randomly selected from the school list and they were invited to participate in the longitudinal project. If one school rejected the invitation, an alternate school randomly selected from the same district was further invited. The process stopped until the selected school accepted the invitation. Finally, 28 schools were successfully recruited to participate in the project between 5 October 2009 and 9 February 2010. Grade 7 students in these schools were invited to complete a battery of questionnaires between 17 November 2009 and 8 July 2010 (i.e., Wave 1) and one year later (i.e., Wave 2). Before students’ participation, consent of their parents was collected through schools via parental notice.

At the two waves, 3,328 (*M*age = 12.59 years, *SD* = 0.74 at Wave 1; 47.60% girls) and 3,638 students (*M*age = 13.64 years, *SD* = 0.75 at Wave 2; 47.84% girls) completed the same questionnaires in their classrooms, respectively, with the presence of two well-trained research staff. Among these students, 2,905 (*M*age = 12.57, *SD* = 0.72 at Wave 1; 49.54% girls) had data at both waves. All the cases were included in the analysis.

The project was approved by the “Human Subjects Ethics Subcommittee” at the authors’ university. Before data collection, the participating schools, adolescent participants, and their parents provided written consent.

### Measures

This study used the 44-item CPYDS-SF. The 80-item long version of the scale included 15 subscales assessing the respective 15 PYD constructs proposed by Catalano et al. [14]. All items were rated from 1 (“*strongly disagree*”) to 6 (“*strongly agree*”). This long version has been validated among Chinese adolescents in Hong Kong and a 4-higher-order-factor model was supported [45]. The short version was formed based on Shek and Ma’s [45] study by selecting three items from each subscale that possessed the highest factor loadings, except for the “self-efficacy” subscale, in which the two items in the long version were included in the short version.

### Data analysis

Data analysis was performed using Mplus 8.5 [52]. First, we checked whether the assumption of multivariate normality was fulfilled. Results revealed normal distributions (|skewness| < 2.0 and |kurtosis| < 7) of all item scores. Thus, the maximum likelihood (ML) estimation method can be correctly used in next steps [53]. Furthermore, the “full information maximum likelihood estimation” incorporated in Mplus was adopted to handle missing values in variables by making full use of all available data [54].

Second, confirmatory factor analysis (CFA) was used to test the conceptual structure of the 44-item CPYDS-SF. We compared two competing models at each wave including the 15-primary-factor structure and the 4-higher-order-factor structure identified by Shek and Ma [45]. Adequate absolute model fit was indicated by multiple indices and criteria [55]: “Comparative Fit Index” (CFI ≥ 0.90), “Tucker-Lewis Index” (TLI ≥ 0.90), “Root Mean Square Error of Approximation” (RMSEA ≤ 0.08), and “Standardized Root Mean Square Residual” (SRMR ≤ 0.08). As Chi-square difference tests may produce bias in a large sample [56], this study used differences in “Bayesian information criterion” (BIC) between the two competing models to decide which model was preferred [57]. The applied cut-off is 10 points (i.e., ΔBIC = 10). Specifically, ΔBIC = 10 indicates a 150:1 likelihood (*p* < .05) and ΔBIC greater than 10 represents “*very strong*” evidence favoring the model with a smaller BIC [58]. This method has been applied in previous research on scale validation [33].

Third, to determine whether the CPYDS-SF measures PYD qualities with the same structure between boys and girls (i.e., across gender) and across the two waves (i.e., over time), invariance tests across gender at each wave and across the two waves were further performed based on the favored factor structure identified in the second step. Following previous practices [33, 59], invariance across gender or overtime was tested sequentially, including (a) configural invariance (free estimation across gender or overtime), (b) metric invariance (equal factor loadings), (c) scalar invariance (equal factor loadings plus equal item intercepts), and (d) strict invariance (equal factor loadings and item intercepts plus equal residual variances). Changes in CFI and RMSEA (i.e., ΔCFI < 0.01 and ΔRMSEA < 0.015) were used to indicate model invariance [60, 61].

## Results

### Comparisons of the two competing structural models

As shown in Table 2, the 15-primary-factor structure (Model 1) demonstrated adequate model fit at the two assessment points (CFI and TLI > 0.90; SRMR and RMSEA < 0.08) while the 4-higher-order-factor structure (Model 2) showed relatively worse model fit as far as CFI and TLI were concerned (i.e., lower than 0.90). In addition, Model 1 showed a lower value of BIC than Model 2 across waves (ΔBIC = 1808.93 and 2987.66 at the two waves, respectively). Thus, the 15-primary-factor structure was retained and used for the subsequent invariance tests. Factor loadings on the 15 dimensions ranged between 0.60 and 0.85 with an average factor loading of 0.72 at Wave 1. At Wave 2, factor loadings ranged between 0.61 and 0.87 with an average factor loading of 0.75. In addition, the 15 dimensions as well as the whole scale showed adequate internal consistency and medium to high inter-factor cross-sectional correlations at both waves (see Table 3). In addition, each of the 15 dimensions also showed a moderate cross-time corrleation with the respective score after one year (*r* = 0.50–0.69, *p*s < .001).

**Table 2:** Summary of model fit for different CFA models at Wave 1 and Wave 2.

Model	Description	*χ* ^*2*^	*df*	CFI	TLI	SRMR	RMSEA (90% CI)	BIC	Δ*χ*^*2*^	ΔCFI	ΔBIC
Wave 1 (*n* = 3,328)											
1	15 primary factor model	6,817.09	793	.92	.91	.04	.054 (.047, .049)	381,726.95			
2	4 higher-order model	9,307.27	877	.89	.89	.05	.048 (.053, .055)	383,535.88	2,490.18	–.03	1,808.93
Wave 2 (*n* = 3,638)											
1	15 primary factor model	7,523.99	793	.93	.92	.04	.048 (.047, .049)	398,631.70			
2	4 higher-order model	11,200.36	877	.89	.88	.05	.057 (.056, .058)	401,619.35	3,676.37	–.03	2,987.66

*Note*. *df* = degree of freedom, CFI = comparative fit index; TLI = Tucker-Lewis index, SRMR = standardized root mean square residual, BIC = Bayesian information criterion, RMSEA = root mean square error of approximation, CI = confidence interval, Δχ^2^ = change in χ^2^, ΔCFI = change in CFI, ΔBIC = change in BIC.

**Table 3:** Reliability of the Chinese Positive Youth Development Scale and correlations among factors at two waves.

Factor	Reliability		Descriptive		Correlations between factors at Wave 1 and Wave 2														
	α	MIIC	Mean/SD																
	W1/W2	W1/W2	W1	W2	1	2	3	4	5	6	7	8	9	10	11	12	13	14	15
1. BO	.74/.77	.49/.53	4.70/.88	4.59/.90	–	.69	.63	.86	.63	.54	.59	.59	.55	.53	.56	.54	.78	.72	.59
2. RE	.79/.81	.55/.59	4.64/.91	4.57/.90	.65	–	.66	.62	.69	.73	.72	.64	.69	.65	.67	.71	.68	.66	.64
3. SC	.86/.88	.66/.71	4.74/.89	4.67/.89	.65	.60	–	.59	.70	.65	.67	.58	.61	.53	.70	.58	.63	.51	.53
4. PB	.76/.77	.52/.53	4.33/.98	4.20/.95	.84	.61	.65	–	.68	.58	.60	.62	.56	.57	.59	.54	.80	.72	.50
5. EC	.73/.74	.47/.49	4.26/.95	4.27/.91	.64	.73	.71	.70	–	.85	.86	.75	.71	.63	.70	.66	.70	.68	.51
6. CC	.81/.83	.59/.62	4.32/.91	4.34/.86	.52	.70	.64	.57	.87	–	.90	.73	.76	.68	.70	.70	.65	.60	.46
7. BC	.71/.76	.45/.52	4.54/.84	4.49/.82	.59	.72	.68	.65	.83	.85	–	.80	.81	.69	.72	.71	.70	.66	.50
8. MC	.73/.75	.48/.50	4.37/.91	4.38/.87	.60	.67	.57	.66	.75	.73	.80	–	.78	.67	.64	.67	.73	.80	.45
9. SD	.75/.78	.50/.55	4.47/.89	4.41/.89	.54	.67	.65	.57	.71	.76	.78	.77	–	.84	.84	.81	.69	.61	.50
10. SE	.65/.68	.48/.51	4.35/.93	4.33/.93	.51	.66	.55	.55	.64	.69	.67	.69	.82	–	.87	.81	.73	.63	.48
11. CPI	.78/.80	.54/.57	4.08/1.03	4.06/1.01	.56	.65	.71	.62	.68	.71	.71	.67	.83	.83	–	.96	.78	.57	.61
12. BF	.84/.85	.64/.66	4.38/1.06	4.26/1.05	.54	.71	.59	.55	.63	.66	.67	.68	.76	.73	.89	–	.82	.64	.59
13. PI	.80/.82	.57/.59	4.37/1.04	4.26/.99	.73	.65	.64	.74	.68	.60	.65	.65	.63	.61	.73	.71	–	.95	.64
14. PN	.72/.72	.46/.47	4.64/.96	4.50/.95	.59	.62	.47	.57	.57	.54	.59	.69	.53	.55	.54	.61	.75	–	.57
15. SP	.88/.89	.71/.73	4.45/1.10	4.32/1.08	.61	.65	.52	.54	.54	.49	.51	.50	.51	.48	.64	.60	.63	.51	–
Total	.96/.96	.37/.38	4.44/.70	4.38/.69															

*Note*. All parameters were significant in correlations (*p* < .001). Parameters below the diagonal represent correlations at Wave 1 and the above ones show correlations at Wave 2. MIIC = mean inter-item correlation, SD = standard deviation, W1 = Wave 1, W2 = Wave 2, BO = boding, RE = resilience, SC = social competence, PB = recognition for positive behavior, EC = emotional competence, CC = cognitive competence, BC = behavioral competence, MC = moral competence, SD = self-determination, SE = self-efficacy, CPI = clear and positive identity, BF = beliefs in future, PI = prosocial involvement, PN = prosocial norms, SP = spirituality.

### Invariance tests

Results of invariance tests across gender are presented in Table 4. The structure with 15 primary factors showed acceptable model fit in both gender groups at the two waves. Sequential invariance tests revealed that changes in CFI and RMSEA were less than the cut-offs (i.e., ΔCFI < 0.01 and ΔRMSEA < 0.015) in all pairs and comparisons. The results implied strict invariance characterized by equal factor loadings, item intercepts, and residual variances of the 15 factors across boys and girls with adequate model fit indices (Wave 1: *χ*^*2*^_*(1688)*_ = 9107.10, CFI = 0.91, TLI = 0.90, RMSEA = 0.05; Wave 2: *χ*^*2*^_*(1688)*_ = 9509.60, CFI = 0.92, TLI = 0.91, RMSEA = 0.05).

**Table 4:** Invariance tests across gender at two waves and longitudinal invariance tests over time.

		*χ* ^*2*^	*df*	CFI	TLI	SRMR	RMSEA (90% CI)	Compare	Δ*χ*^*2*^	ΔCFI	Δ*df*	ΔRMSEA
Invariance tests across gender at Wave 1												
	Boys (*n* = 1,740)	3,777.80	793	.93	.91	.03	.047 (.045, .048)					
	Girls (*n* = 1,584)	4,334.25	793	.91	.89	.04	.053 (.052, .055)					
	A. Configural	8,112.06	1,586	.92	.90	.04	.050 (.049, .051)					
	B. Metric	8,170.18	1,615	.92	.90	.04	.049 (.048, .050)	B vs. A	58.12	.000	29	–.001
	C. Scalar	8,433.21	1,644	.92	.90	.04	.050 (.049, .051)	C vs. B	263.03	–.003	29	.001
	D. Strict	9,107.10	1,688	.91	.90	.05	.051 (.050, .052)	D vs. C	673.89	–.008	44	.001
Invariance tests across gender at Wave 2												
	Boys (*n* = 1,895)	4,231.47	793	.93	.92	.03	.048 (.046, .049)					
	Girls (*n* = 1,739)	4,347.04	793	.92	.90	.04	.051 (.049, .052)					
	A. Configural	8,578.51	1,586	.93	.91	.04	.049 (.048, .050)					
	B. Metric	8,697.74	1,615	.93	.91	.04	.049 (.048, .050)	B vs. A	119.23	–.001	29	.000
	C. Scalar	8,970.22	1,644	.92	.91	.04	.050 (.049, .051)	C vs. B	272.49	–.003	29	.001
	D. Strict	9,509.60	1,688	.92	.91	.05	.050 (.050, .051)	D vs. C	539.38	–.005	44	.000
Invariance tests over time (*n* = 4,061)												
	Wave 1 (*n* = 3,328)	6,817.09	793	.92	.91	.04	.048 (.047, .049)					
	Wave 2 (*n* = 3,638)	7,523.99	793	.93	.92	.04	.048 (.047, .049)					
	A. Configural	17,028.77	3,253	.93	.91	.03	.032 (.032, .033)					
	B. Metric	17,100.94	3,282	.93	.91	.03	.032 (.032, .033)	B vs. A	72.17	.000	29	.000
	C. Scalar	17,453.52	3,311	.92	.91	.03	.032 (.032, .033)	C vs. B	352.58	–.002	29	.000
	D. Strict	18,119.54	3,355	.92	.91	.04	.033 (.032, .033)	D vs. C	666.02	–.003	44	.001

*Note*. *df* = degree of freedom, CFI = comparative fit index; TLI = Tucker-Lewis index, SRMR = standardized root mean square residual, RMSEA = root mean square error of approximation, CI = confidence interval, Δχ^2^ = change in χ^2^, ΔCFI = change in CFI, Δ*df* = change in df, ΔRMSEA = change in RMSEA.

Results of longitudinal invariance tests over the two waves are also shown in Table 4. As changes in CFI and RMSEA were less than 0.01 and 0.015, respectively, in all pairs of model comparisons, longitudinal invariance of the 15-primary-factor structure was supported. The strict invariance model (i.e., equal factor loadings, item intercepts, residual variances over time) showed adequate model fit (*χ*^*2*^_*(3355)*_ = 18119.54, CFI = 0.92, TLI = 0.91, RMSEA = 0.03).

To sum up, the above findings supported across-gender and longitudinal invariance of the 15-primary-factor structure of the 44-item CPYDS-SF.

## Discussion and conclusions

The growing interest among researchers and practitioners in positive youth development (PYD) frameworks highlights the necessity for developing effective PYD assessment tools that are psychometrically sound, empirically helpful, and culturally applicable. Nevertheless, findings from studies validating PYD measures among adolescents in Western contexts may not be entirely generalizable to non-Western settings, such as Chinese contexts. This research gap has been partially addressed by previous research that examined the validity and reliability of the 80-item long form of the “Chinese Positive Youth Development Scale” (CPYDS-LF). The research supported the scale’s four-higher-order-factor structure and its invariance across gender groups [45]. The present study further addressed the aforementioned research gap by investigating factorial validity of the 44-item short form of the scale (CPYDS-SF) and measurement invariance across gender groups and over time among secondary school students in Hong Kong. Results showed that the CPYDS-SF is best represented by the 15-primary-factor structure (e.g., “social competence,” “moral competence,” and “resilience”) rather than the four-higher-order-factor structure (e.g., “cognitive-behavioral competence” and “prosocial attributes”). Invariance tests provided support for factorial invariance of the 15-primary-factor structure across gender and over time. These findings provide support for the original conceptual model of the CPYDS.

The 80-item CPYDS-LF consists of 15 subscales that can be further subsumed into four higher-order factors. CPYDS-SF was formed by extracting items with the highest factor loadings in each subscale of CPYDS-LF. This approach has been commonly used in forming a short version of a measurement tool [62, 63]. This is because extracting items with the highest factor loadings can improve parsimoniousness while maintaining a high level of validity of the scale [64]. However, in the CPYDS-SF, the 15-primary-factor structure fitted the data better than the four-higher-order-factor structure. One explanation is that the retention of some specific items may reduce the common variance among items in different primary dimensions [65], thus making it difficult to give support for the higher-order factors. It is worth noting that items in the CPYDS-SF showed medium to high factor loadings and subscales showed adequate psychometric properties, which is consistent with features of other validated PYD measures [21, 22]. Therefore, CPYDS-SF is well represented by the 15-primary-factor structure.

Gender differences in PYD research fields have been a common topic of concern. For example, among the “Five Cs,” Gomez-Baya et al. [40] found that boys scored higher in “*competence*” and “*confidence*”, whereas girls scored higher in “*connection*,” “*caring*,” and “*character*”. To correctly interpret such gender differences in terms of multiple possibilities, such as gender roles, educational systems, and cultural factors [66, 67], measurement invariance across gender should be first established to rule out the possibility of different measurement structures between girls and boys. Furthermore, measurement invariance of PYD assessment tools across gender is essential to correctly interpret gender differences in the effects of youth enhancement programs [67–69]. The present study found that the 15-primary-factor structure of the CPYDS-SF was invariant across gender groups. This is in line with the findings of measurement invariance for the CPYDS-LF and other PYD measures [e.g., 22, 26, 70] among Asian samples. These findings cumulatively indicate that boys and girls tend to interpret PYD measurement items in the same way. Thus, gender differences in PYD qualities and program effectiveness can be attributable to developmental differences rather than measurement differences between gender groups.

In addition to gender differences, it is also a common practice that PYD research investigates longitudinal development of adolescents’ PYD attributes [71, 72]. Adolescence is associated with rapid biological, neurological, and cognitive development [35]. For example, enlargement of the ventromedial prefrontal cortex enables adolescents to have better abstract thinking and develop autonomy and independence [73, 74]. Due to these developmental changes, adolescents’ perceptions and interpretations of themselves (e.g., their physical appearance, competence, and relationships) and things around them have been commonly found to change during the adolescent period [75, 76]. However, as Hoyle and Smith [77] pointed out, the comparison across time points without longitudinal invariance measurement is a classic example of “comparing apples and oranges” (p. 433). In other words, we can only “promote a more thorough and unambiguous understanding of developmental stability and change” [78, p. 287] if “the invariance holds longitudinally” [79, p. 5]. As a result, examining longitudinal measurement invariance is a prerequisite to ensure that any difference shown in PYD qualities over time represents actual developmental changes rather than adolescents’ different interpretations of assessment items [42, 80]. In this regard, the strict longitudinal invariance of the CPYDS-SF ensure that the scale measures the same PYD constructs on different occasions, enabling correct and meaningful investigations of longitudinal development or age group differences in PYD qualities among Chinese adolescents.

The present study has important implications in child and adolescent research. First, validation of the CPYDS-SF suggests that the short version can be effectively used in measuring Chinese adolescents’ PYD qualities, shortening the time to fill in the questionnaire while also maintaining the validity and reliability of the measurement. This is much more likely to enhance schools’ and adolescents’ willingness to take part in and reduce participants’ fatigue, especially considering that Chinese adolescents tend to have heavy schoolwork [81]. Second, the CPYDS-SF with strict gender and time invariance provides researchers with a valid and reliable assessment tool to investigate the development of PYD qualities, its antecedents (e.g., parental influence) as well as its contribution to adolescent developmental outcomes (e.g., academic and emotional well-being). Third, the CPYDS-SF also serves as a useful assessment tool for evaluating the effectiveness of PYD programs in Chinese communities. Specifically, scores obtained from the CPYDS-SF before and after program completion or between students participating in or not can be meaningfully compared to delineate potential impacts of program participation. Noteworthy, the long version of the scale has been successfully utilized in such program evaluation in both Hong Kong and mainland China [11, 82]. The current study further supports the similar employment of the short version in program evaluation.

Despite the pioneering nature of the study in the PYD literature, the present study has several limitations. First, only the data based on Grade 7 students over two waves were used. To portray a more holistic picture of the psychometric properties of the CPYDS-SF, adolescents from different age groups (e.g., students from senior high schools) can be further recruited in future studies. Second, the current sample only included adolescents in Hong Kong, thus making the present findings might not fully applicable to adolescent samples in other Chinese communities, such as different areas (e.g., rural and urban) in mainland China. Thus, future research is suggested to test the factor structure of the CPYDS-SF and its invariance in different adolescent samples. Third, the present study only validated the CPYDS-SF among adolescents. As PYD scales have also been widely utilized among emerging adults such as university students [69, 83], future studies will benefit from further validating CPYDS-SF among Chinese university students so that the usage of the scale can be extended. Fourth, the present study utilized data collected several years ago. Due to the rapid social and environmental changes, including the influence of COVID-19, adolescents’ perceived PYD attributes may change as well. Thus, there is a need for replication using more recent data, which is important to ensure the stability and generalizability of the present findings and support the use of the CPYDS-SF in future studies. Nevertheless, the present studt can still give us a snapshot on the factorial invariance of the CPYDS-SF. Lastly, as we focused on examining the factorial validity and factorial invariance of the scale, we did not examine test-retest reliability, which is also an important factor of the scale’s psychometric properties. While individual dimensions in the CPYDS-SF demonstrated moderate cross-time correlations after one year, which can be regarded as support for test-retest reliability, futhre studies will certainly benefit from directly investigating the test-retest reliability.

Despite these limitations, findings in this study suggest that Hong Kong Chinese adolescents’ manifestation of PYD qualities assessed by the CPYDS-SF was well represented by the 15 primary factors proposed by Catalano et al. [14]. In addition, the current 15-primary-factor model was strictly invariant between male and female adolescents over one year, implying that the factor structure of PYD qualities was equivalent between gender groups and stable over time. To conclude, the presenting findings suggest that the CPYDS-SF can be reliably utilized in investigating gender differences and longitudinal changes in PYD qualities among Chinese adolescents.

## Supporting information

S1 File All data underlying the present findings can be found in S1 File. (XLSX)
